# Uropathogenic *E*.*coli* (UPEC) Infection Induces Proliferation through Enhancer of Zeste Homologue 2 (EZH2)

**DOI:** 10.1371/journal.pone.0149118

**Published:** 2016-03-10

**Authors:** Kenneth Ting, Karen J. Aitken, Frank Penna, Alaleh Najdi Samiei, Martin Sidler, Jia-Xin Jiang, Fadi Ibrahim, Cornelia Tolg, Paul Delgado-Olguin, Norman Rosenblum, Darius J. Bägli

**Affiliations:** 1 Faculty of Arts and Sciences, University of Toronto, Toronto, Ontario, Canada; 2 Institute of Medical Sciences, Faculty of Medicine, University of Toronto, Toronto, Ontario, Canada; 3 Developmental and Stem Cell Biology Program, Research Institute, Hospital for Sick Children, Toronto, Ontario, Canada; 4 Urology Division, Department of Surgery, Hospital for Sick Children, Toronto, Ontario, Canada; 5 Physiology and Experimental Medicine, Research Institute, Hospital for Sick Children, Toronto, Ontario, Canada; 6 Nephrology Division, Department of Surgery, Hospital for Sick Children, Toronto, Ontario, Canada; University of Illinois at Chicago College of Medicine, UNITED STATES

## Abstract

Host-pathogen interactions can induce epigenetic changes in the host directly, as well as indirectly through secreted factors. Previously, uropathogenic *Escherichia coli* (UPEC) was shown to increase DNA methyltransferase activity and expression, which was associated with methylation-dependent alterations in the urothelial expression of CDKN2A. Here, we showed that paracrine factors from infected cells alter expression of another epigenetic writer, EZH2, coordinate with proliferation. Urothelial cells were inoculated with UPEC, UPEC derivatives, or vehicle (mock infection) at low moi, washed, then maintained in media with Gentamycin. Urothelial conditioned media (CM) and extracellular vesicles (EV) were isolated after the inoculations and used to treat naïve urothelial cells. EZH2 increased with UPEC infection, inoculation-induced CM, and inoculation-induced EV vs. parallel stimulation derived from mock-inoculated urothelial cells. We found that infection also increased proliferation at one day post-infection, which was blocked by the EZH2 inhibitor UNC1999. Inhibition of demethylation at H3K27me3 had the opposite effect and augmented proliferation. CONCLUSION: Uropathogen-induced paracrine factors act epigenetically by altering expression of EZH2, which plays a key role in early host cell proliferative responses to infection.

## Introduction

For many patients with urinary tract infection (UTI), primary infection leads to chronic or recurrent infections without a strong genetic association. The overall prevalence of UTI is quite high, accounting for 1% of all doctor’s visits in the USA. Its prevalence is high across many populations: 3–8% of female children [1,2] and 50–60% of adult women [3,4] will have a UTI in their lifetime, with a global incidence of ~150 million cases per year (for recent reviews see [5,6]. It is also the most common nosocomial and catheter-associated infection. These acute episodes are often followed by chronic or recurrent UTI (rUTI) in 30–50% of female children and 25% of adult women with prior acute UTI with increasing rates of antibiotic resistance (for reviews see: [6,7]).

Acute infection leads to cell death and subsequent regenerative proliferation of the intermediate cells to reform the critical urothelial barrier[8]. After this initial wave of infection, uropathogenic *E*.*coli* (UPEC) can escape the host immune response, become dormant and form quiescent intracellular bacterial reservoirs (QIR), which persist chronically inside uroepithelial cells [9]. Recurrent UTI include *relapses* (symptomatic infections with the same organism following therapy) and *reinfections* (different bacterial isolate or previously isolated bacteria after treatment with negative urine culture). Recurrent UTI may be associated with reactivation of the QIR. As intracellular UPEC inoculation *in vitro* can induce the host epigenetic machinery (e.g. DNMT1) alongside alterations in gene expression, the potential exists to alter host cell responses to the bacteria [10,11]. An examination of the dynamic changes of other epigenetic writers during the initial wave of infection may help uncover the role of these writers in mediating both beneficial and deleterious response of the host cell to UTI.

Epigenetic changes can be defined as structural changes in the packaging of chromosomal regions which can perpetuate ongoing alterations in gene or protein expression or activity states[12]. Epigenetic enzymes are categorized as readers, writers and erasers of the epigenetic marks and can induce both persistent and dynamic changes[13]. DNA methyltransferases (DNMTs) and Enhancer of Zeste Homologue 2 (EZH2) are epigenetic writers that catalyse changes onto the CpG DNA or histone tails, respectively. Cells use epigenetic alterations to modulate gene or protein responses to the environment irrespective of primary gene sequences, often resulting in altered phenotype. In addition, the epigenetic machinery can respond rapidly to alterations in the environment[14], including the pathologic and symbiotic microbes around and inside the host cell[11]. Due to the greater reversibility through histone demethylases, histone methylation allows for more dynamic changes than DNA methylation.

UPEC induce obvious changes in the host cell, including apoptosis and inflammatory signaling, as well as epigenetic alterations[11,15–18]. Intracellular pathogen subversion of host epigenetics has been reported to occur through CpG DNA, histone modifications and non-coding RNAs [11,19–26]. However, there are few studies exploring if bacteria alter expression of host epigenetic writers. With regard to uropathogenic intracellular bacteria, two studies show that UPEC alters activity or expression of major epigenetic writers, DNMT1 and histone acetylases [11,27]. Specifically, UPEC inoculation increased expression of host DNMT1, which was inversely associated with alterations in expression of CDKN2A [10,11]. However, many other epigenetic regulators of CDKN2A exist, including EZH2. EZH2 has a well known function in cellular proliferation[28,29] through its modulation of H3K27me3 marks at promoters of tumour suppressors, e.g. CDKN2A genes, and alteration of pro-proliferative protein activity. EZH2’s regulatory role in stem cells is clear in epithelial, mesenchymal and pluripotent stem cells[30–35]. In another infection model, *Citrobacter* spp. intestinal infection increases EZH2’s repression of a Wnt-repressive factor, WIF1 (Wnt inhibitory factor 1) coincident with crypt hyperplasia[36]. Interestingly, epigenetic repression of WIF1 also occurs during urinary tract infection induced by schistosomes [37]. As WIF1 binds to WNT proteins, the loss of WNT can increase WNT/beta-catenin signaling.

During other *in vivo* and *in vitro* infections, host mRNA expression can be subverted by secreted factors from neighboring infected cells [38–42]. A wide variety of secreted molecules have been found in conditioned media and EV from infected cells. Particularly, diverse miRNAs in secreted extracellular vesicles (EVs) are released from infected cells and tissues, having broad epigenetic effects on neighbouring naive cells[3,38,42] and antibacterial defense [3].

In this study, we examined whether the most common intracellular bacterial pathogen of the urinary tract can increase expression of a critical regulatory component of the epigenetic machinery (EZH2). We determined that UPEC-induced secretion of host factors that lead to elevated nuclear expression of EZH2. Importantly, the secreted factors included release of extracellular vesicles (EV), which also had the ectopic ability to induce EZH2 expression. Moreover, pharmacologic inhibition of EZH2 activity led to a decrease in infection-induced proliferation at the 24-hour time period. Pathogen-induced paracrine factors appear have crucial roles in regulating the epigenetic and proliferative repertoire of host cells.

## Materials and Methods

### Bacterial Strains and Cell Lines

UPEC-derived strains, (SLC2-33-1 and SLC2-35-1), and UPEC parent strain UTI89, were graciously provided by Dr. Scott Hultgren (Washington University, St Louis, USA) [43]. The strain SLC2-33-1 (FIM H^+^) has the wildtype Fim H fimbriae structure, enabling intracellular infection. The latter strain (SLC2-35-1, FIM H^**Δ**^) lacks the wildtype Fim H structure due to a point mutation, decreasing its ability to invade host cells. Bacteria were inoculated into LB broth from single colonies of LB agar plates. They were grown in static culture at 37°C overnight, then re-inoculated into 150 mL cultures for 4–6 hours, 37°C. OD600 readings of serial dilutions of bacteria were compared to a standard curve derived from serial dilutions that were spread on LB agar. Growth of SLC strains in LB broth was performed with Kanamycin (25 μg/mL). The HT-5637 cell line was obtained from American Type Culture Collection (ATCC, Manassas, VA, USA), is used to model intracellular UPEC infection[11].

### In vitro infection

Host urothelial cells (HT-5637) were cultured for 48 hours prior to inoculation at a density of 100,000 cells/ml with RPMI-1640 (1X), 5% Fetal Calf Serum (FCS) and 1% (100X) Antibiotics/Antimycotic (all purchased from MultiCell Technologies, Woonsocket, RI, USA), in a humid environment at 37°C and 5% CO_2_. Primary urothelial cells from ScienCell (Carlsbad, CA) were maintained in serum-free Urothelial Cell Media plus additives (ScienCell).

Prior to inoculations, cells were transferred to starvation media RPMI-1640 (1X) without antibiotic for 2–4 hours. SLC2-33-1 (FIM H^+^) and SLC2-35-1 (FIM HΔ) or parent strain UTI89, were grown in broth overnight, and subsequently quantified against a standard curve with a spectrophotometer at O.D. 600. Cells were inoculated with FIM H^+^ and FIM HΔ respectively at the optimized multiplicity of infection (MOI) of 2 *E*.*coli* per cell for 2 hours. After 2 hours of inoculation time, HT-5637 cells were washed with PBS (1X) containing 0.1% Gentamycin (50 ug/ml) (MultiCell Technologies) every 5 minutes for three times to inhibit growth of bacteria in media outside host cells. HT-5637 cells were incubated with RPMI-1640 (1X), 5% FCS and 0.02% Gentamycin (50 ug/ml) for the times indicated for each experiment (up to 3 weeks) post-inoculation (p.i).

### Immunofluorescence of H3K27me3, EZH2 and PCNA

Cells (HT-5637) were plated at a density of 100,000 cells/ml into 4-well chambered slides or 24 well plates (Thermo Fisher Scientific, Mississauga, Canada) with RPMI-1640, 5% FCS and Antibiotics/Antimycotic, in a moistened environment at 37°C and 5% CO_2_, for 48 hours prior to inoculation. Cells were inoculated as described above. At 24 hours p.i., cells were fixed in 4% paraformaldehyde in PBS, 15 minutes at room temperature, and washed three times with PBS for 5 minutes. Cells were incubated in PBS with 0.2% Triton X, 15 minutes, and later washed with PBS for three times, 5 minutes each. Normal donkey serum (10%) in PBS was added as a blocking agent for 30 minutes. As indicated in legends and results, primary antibodies (rabbit anti-H3K27me3, mouse anti-PCNA & rabbit anti-EZH2 (Cell Signal, Boston, MA, US) or mouse anti-EZH2 (Cell Signal)), at 1:200 dilution in 10% normal donkey serum with PBS were added to slides, and incubated at 4°C overnight. Post incubation, cells were washed with PBS for three times, five minutes each, at room temperature. Secondary antibodies, donkey anti-mouse Cy3, anti-rabbit far-red (Jackson ImmunoResearch Laboratories, PA, USA; 1:200) were incubated 10% normal donkey serum with PBS for 60 minutes at room temperature. The cells were washed with PBS and stained with the nuclear dye, Hoechst 33342. Slides were mounted with fluorescence mounting medium (Dako, Burlington, Canada). Immunofluorescence of EZH2 was captured by confocal microscopy through a quorum spinning disk confocal microscope (Olympus 1X81) equipped with a Hamamatsu digital camera in the Imaging Facility of the Sickkids Research Institute. Experiments were performed with replicates as described in the Figure Legends. Mean fluorescence within each cell and nucleus was quantified using Volocity 3D Image Analysis software.

### Conditioned Media preparation and treatment

Conditioned media (CM) were collected after 1 or 2 days, by centrifugation of cell debris and passing twice through 0.2 micron filters. CM for the preparation of extracellular vesicles (EV) were **not** passed through 0.2 micron filters, but were immediately centrifuged to remove cell debris (see below for EV preparation). CM at 50% of total media were added to naive urothelial cells for 2 hours, or 1, 2, 6 days.

### Extracellular Vesicle preparation, analysis and treatment

Exosome-free serum was used for extracellular vesicle (EV) isolations. CM for preparation of exosomes was **not** passed through 0.2 micron filters; instead it was used immediately for EV isolation, which included an initial centrifugation of bacteria and cell debris. EV were isolated from fresh CM with ExoQuick TC (System Biosciences). EV were prepared for Nanosight diluting 0.1 mL exosomes into 1 mL double-distilled sterile water. Transmission EM was performed at the TEM facility of the Mt. Sinai Hospital, Toronto, Canada, using their exosome protocol as follows. Exosome pellets were resuspended in 2% paraformaldehyde, and deposited on Formvar-carbon coated EM grids, 20 minutes. Exosomes on grids were transferred into an equal volume of PBS and then 0.25 volume of 1% gluteraldehyde 5 min. Grids with exosomes were washed in distilled water seven times. Samples were contrasted with uranyl-oxalate (UA), pH 7, 5 minutes and embedded in methyl cellulose-UA, 10 minutes on ice. Grids were removed with stainless steel loops, blotted, air-dried and observed under electron microscope at 80 kV. EV treatment of naïve cells was performed by adding EV derived from an equivalent of 0.5 starting CM volume to naïve urothelial cells for 2 days. In the EV labeling experiment, EV were labeled with a green fluorescent-channel membrane labeling kit (Active Motif) and re-purified with ExoQuick overnight to remove excess label before addition to cells. Experiments were performed in triplicate for TEM, and 5 replicates for EV fluorescence.

### mRNA expression analysis

RNA was isolated using Trizol according to standard methodologies (Aitken et al, 2010[44,45]). Reverse transcription was performed with SuperScript III (Life Technologies; as in Jiang et al, 2013[11,14,45]). As in Aitken *et al*, 2010, we performed QPCR using exon-spanning primers designed on Primer3 and checked on PrimerBlast (ncbi). These primers (S1 Table) were designed to include commonly expressed isoforms of each mRNA species. QPCR experiments were performed in replicates of 5.

#### Viability assays

For viability assays, 20μl of resazurin dye from the *In vitro* Toxicology Assay Kit (Sigma-Aldrich, Oakville, ON, Canada) was then added to each well and the cells were further incubated at 37°C for another 2 hours. Wells with media and resazurin dye was used for negative controls. Upon 2 hours of incubation, the fluorescence of the media solution was subsequently quantified with the fluorescent plate reader (Molecular Devices) with excitation at 560nm, emission and cut off at 590nm. Replicates for viability were 8.

### Histone lysine 27 methylase and demethylase inhibition

Dose response curves were performed to select the appropriate dosage for the HT-5637 cells. HT-5637 were cultured at a density of 20,000 cells/well in a 96 well plate with RPMI-1640 (Wisent), 5% FCS and Antibiotics/Antimycotic (1x, Wisent), in a humidified environment at 37°C and 5% CO_2_, for 48 hours. To maintain conditions similar to the inoculation protocol, cells were serum-starved in media was changed to RPMI-1640 (1X) without serum overnight. Upon inhibitor treatment, cells were cultured in RPMI-1640 (1X), 5% FCS, Gentamycin (50mg/ml), in dilutions of UNC1999 [46,47] or GSKJ4[48,49], for 24 hours. A Dose-finding assay with the resazurin viability assay determined that 500nM UNC1999 and 100nM GSKJ4 (Sigma-Aldrich, Oakville, ON, Canada) were non-toxic and appropriate for the cells in the proliferation experiments (Figs A-C in S1 Fig). Host cells treated with vehicle served as negative controls. The cells inoculated with FIM H^Δ^ *E*.*coli* acted as a control for potential non-infectious bacterial related responses. The replicates for each group were n = 8, with the results of multiple cells averaged for each n.

### Statistics

Student’s *t*-test and Pearson’s correlation analysis was performed to compare expression levels between groups, with *p*<0.05 considered statistically significant.

## Results

### UPEC increases activity and expression of the epigenetic writer, EZH2

**Fig 1:**
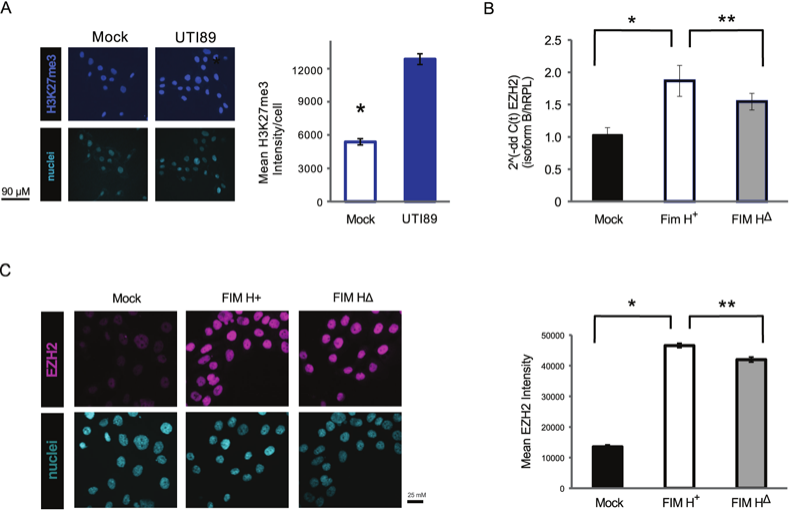
EZH2 activity and expression in HT-5637 urothelial cells is increased by in vitro inoculation of uropathogenic *E*.*coli* (UPEC). (A) H3K27me3 is a marker of EZH2 activity. H3K27me3 immunostaining in UPEC inoculated urothelial cells (10^5^ cells/ml) was significantly upregulated after 18 hours post-inoculation (p.i.) with UPEC (UTI89). Levels of the histone mark in nuclei were quantified by image analysis of confocal microscopy images. Fluorescence intensity was compared between HTB9 cells +/- UPEC inoculation at 18 hours p.i. (*, p<10^−10^, 2-tailed, student’s t-test). N = 5. (B) Expression of EZH2 isoform b mRNA increased after 18 hours p.i. in the UTI89 Fim H^+^ derivative as well as the point mutant Fim HΔ which is less able to invade host HT-5637 cells. N = 5. (C) Immunofluorescent staining reveals a similar rise in protein expression of EZH2 p.i. in Fim H^+^ inoculated cells. HT-5637 cells were inoculated with UPEC mutant strains complemented with Fim H^+^ or Fim HΔ plasmids. N = 6.

As EZH2 has been implicated in responses to other infections [36,50], we examined if a single uropathogenic infection could alter expression of the critical epigenetic writer, EZH2, and the histone mark which it catalyzes, H3K27me3. Activity of EZH2 can be detected through an immunostaining method for H3K27me3[51]. UPEC inoculation for 2 hours led to higher H3K27me3 signal (a cognate for EZH2 activity) at 24 hours (Fig 1A). A UTI89 Fim H^+^ derivative also significantly increased EZH2 mRNA expression (Fig 1B) and EZH2 nuclear expression at 18 hours post-inoculation (Fig 1C) above control. The Fim H^+^ derivative also showed increased expression compared to a point-mutated derivative, Fim H^**Δ**^, which is less able to invade host cells (Fig 1B and 1C).

### UPEC increases expression of EZH2 through paracrine factors

**Fig 2:**
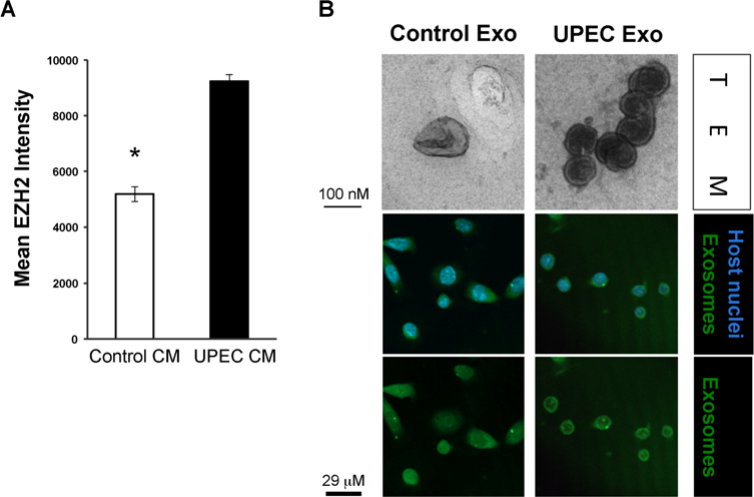
EZH2 expression is increased by paracrine factors from infected cells. (A) Incubation of HT-5637 cells for 48 hours in conditioned media (CM) from UPEC infected or mock-infected control cells increased EZH2 expression by immunofluorescent staining. UPEC- FIM H^+^-induced CM significantly increased EZH2 expression in naïve cells compared to control CM (p<0.005 UPEC vs normal). (B) Intracellular UPEC infection induces secretion of clearly defined extracellular vesicles (EV). EV were isolated from CM using ExoQuickTC (System BioSciences). Top panel: Transmission electron microscopy (TEM) of EV released by cells that had been UPEC- or mock-inoculated with UPEC and cultured for 48 hours in complete media with exosome-free serum and Gentamycin. Immunostaining panel: EV were labeled in green (Active Motif cell membrane kit), re-isolated and then added to Naïve cells for 2 hours. EV-treated cells were washed and fixed in 4% paraformaldehyde. Green fluorescence indicates EV that have fused and in some cases integrated into cells. N = 3. Representative photomicrographs shown.

Previously, we noted that uninfected cells in the inoculated cultures *in vitro* also express higher levels of DNMT1, an epigenetic writer [11]. Therefore, we questioned if infected cells release factors that can initiate a paracrine increase in another epigenetic writer, EZH2, in naïve (uninfected) cells. Exposing naïve host cells to infection-induced CM led to an increase in EZH2 expression in the nucleus (Fig 2A).

Similarly, we examined if extracellular vesicular components released by host cells into the CM might be playing a role in this ectopic response. We isolated extracellular vesicles (EV) from cultured urothelial cells by two methods and found that the ExoQuick methodology performed well for the isolation of EV within the desired characteristic size range (Table 1) by Nanosight. In this approach only 0.4% and 2% of particles were smaller than 30 nm from HT-5637 and normal primary human urothelial cells, respectively. The majority of vesicles fell in the expected size range of >30 nm to <250 nm, with the greatest number of vesicles between 30 and 150 nm in size, with a mean of 111 nm. We examined EV from freshly isolated conditioned media by transmission electron microscopy (TEM) and uranyl oxalate staining. Infection-induced EV were readily identified by TEM as circular cup-shaped exosomes with a lipid bilayer, whereas the control cell EV were more difficult to locate and identify, as they were qualitatively fewer in number and less well defined (Fig 2B). Fusion of labeled EV with the cell membranes led to uptake of the cell membrane label in green (Fig 2B, lower panels).

**Table 1:** Nanosight quantitation of the size and number of extracellular vesicles (EV) from normal primary and cancer cell lines from the urothelium reveal a characteristic size and range of particles for EV. 20% of EV preparations isolated by serial ultracentrifugation had particles <30nm in size, which is indicative of a high level of non-EV debris, indicating that the ExoQuickTC method was appropriate for our studies.

Cell type	Concentration	Max particle size	Peak:	Particle<30 nm
HTB9 (carcinoma cell line)	10^6^/mL	178 nm	111 nm	0.40%
Normal primary urothelial	10^6^/mL	242 nm	129 nm	2%

**Fig 3:**
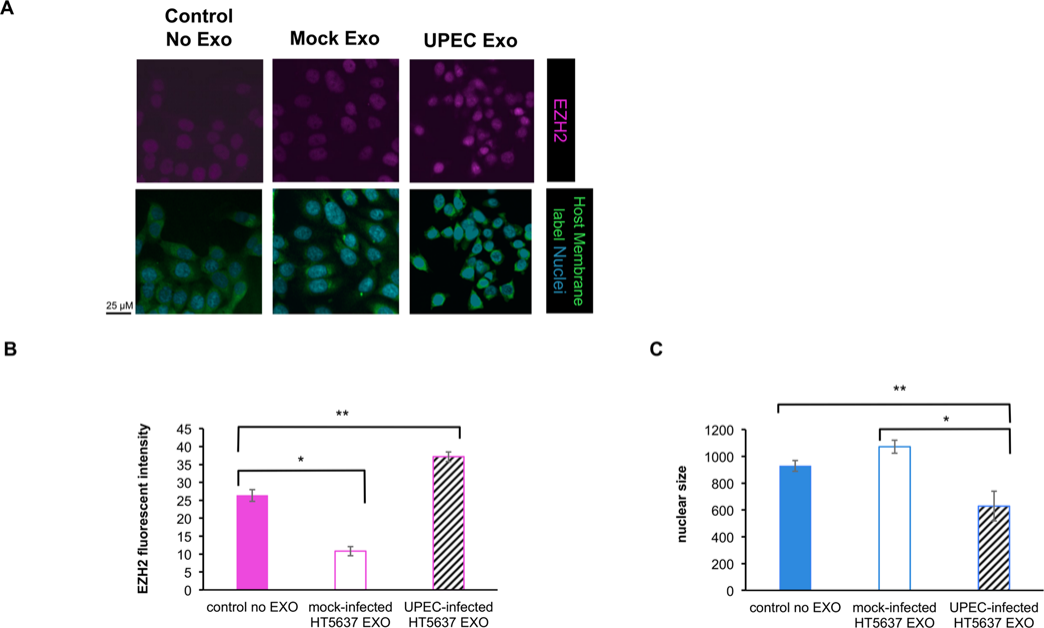
Inoculated HT-5637 urothelial cells produce extracellular vesicles (EV) with epigenetic potential. (A) Unlabelled EV from Fim H^+^ inoculated host cells (vs from mock-infected cells) were able to upregulate epigenetic machinery (EZH2) in naïve urothelial (HT-5637) cells, (p<0.005). (B) EZH2 fluorescent signals were imaged by confocal microscopy and quantitated using Volocity software. (C) Quantitation of nuclear size was performed using Volocity software, based on the area occupying the Hoechst 33342 signal. Fim H^+^ -inoculated host cells had smaller nuclei than control groups. N = 6.

**Fig 4:**
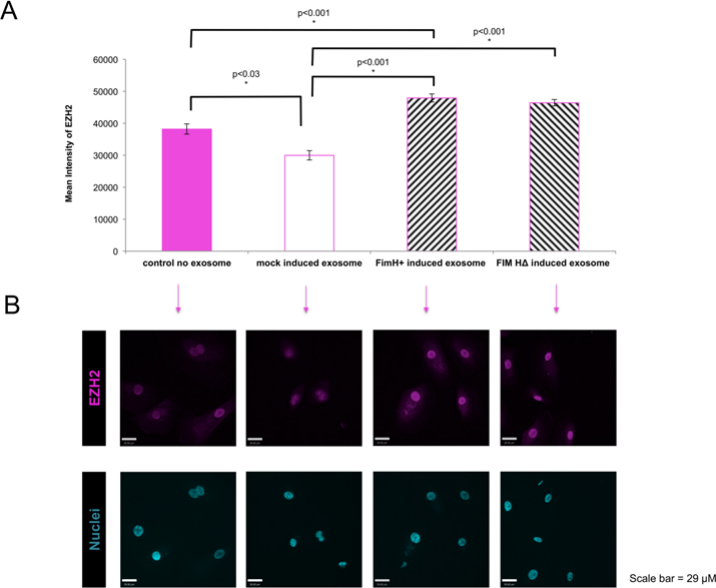
Normal primary human urothelial cells produce extracellular vesicles (EV) with epigenetic potential. (A) Unlabelled EV from Fim H^+^ or Fim HΔ inoculated host cells (vs control EV or mock-infected cells) were able to upregulate epigenetic machinery (EZH2) in naïve primary human urothelial cells, (p<0.005). Some cytoplasmic EZH2 was present in all fields. (B) EZH2 fluorescent signals were imaged by confocal microscopy and quantitated using Volocity software. Exosomes from Fim H^+^ or Fim HΔ inoculated host cells induced upregulation of EZH2, whereas control exosomes caused a decrease in EZH2 expression. N = 6.

Treating naïve cells with EV of infected cells caused an increase in EZH2 expression in both HTB9 and primary urothelial cells (*p*<0.005; Figs 3 and 4). The urothelial cell nuclei appear smaller in the presence of the EV (Fig 3C). Interestingly, EV derived from **control uninfected** primary urothelial cells caused a downregulation of EZH2 expression on naïve recipient primary urothelial cells, compared to control (no exosome-treated) HT-5637 and primary urothelial cells (Figs 3 and 4).

### EZH2 plays a crucial role in the urothelial cell proliferative response to UPEC infection

**Fig 5:**
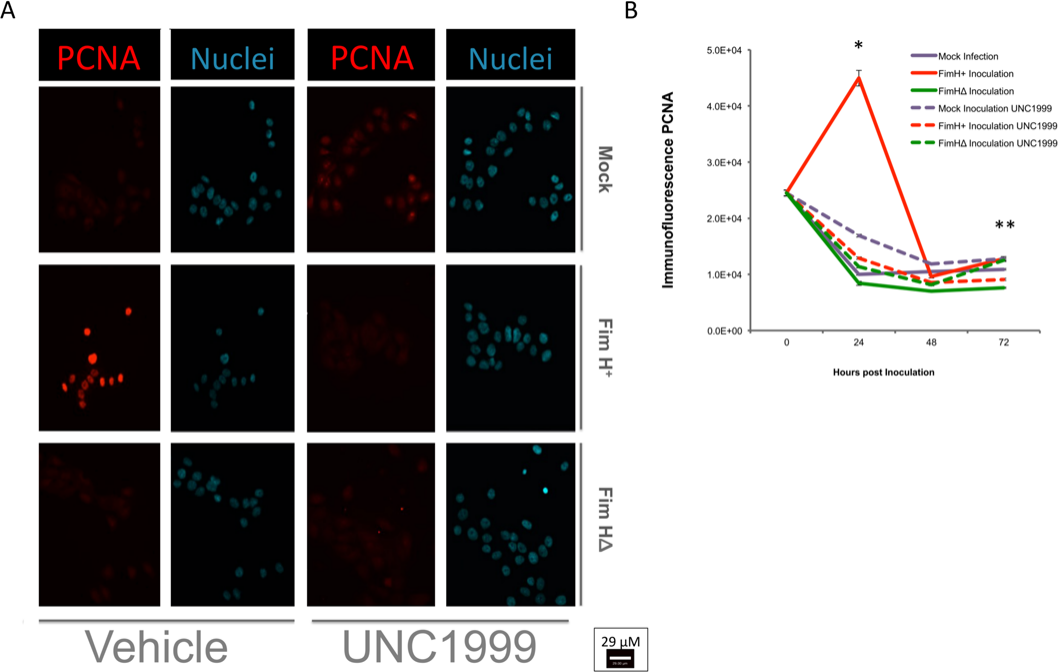
EZH2 plays a crucial role in proliferative response to intracellular infection. After acute infection, the initial wave of cell lysis is followed by a proliferative response peaking at 24 hours post-inoculation. As cell numbers can vary during these phases, proliferation was detected by PCNA staining instead of cell counting. The highly selective inhibitor of EZH2, UNC1999 (500 nM), was able to prevent urothelial proliferation in response to UPEC infection at both 24 hours and 72 hours post-inoculation (p.i.). UPEC Fim H^+^ bacteria increased proliferation in host cells at 24 hours (P<0.000001 vs. other groups) and led to higher proliferative levels above FimHΔ inoculated or UNC1999-treated Fim H^+^ inoculated levels at 72 hours p.i. N = 8. *, p<0.0001, 2-tailed, student’s t-test.

In vivo, urinary tract infection leads to loss of urothelial cells and then in vivo urothelial cell proliferation. We have utilized a model *in vitro* inoculation with UPEC at low moi (1–2), which allows for continued host cell survival and proliferation alongside bacterial persistence (Fig A in S4 Fig and S5 Fig). As EZH2 is known to have a critical role in proliferation[28,52], we examined the function of EZH2 on host cell proliferation after UPEC inoculation. After the 2-hour infection period, followed by washing, the EZH2 selective inhibitor (UNC1999; vs. vehicle) was added to the host cell media containing Gentamycin. We found that UPEC infection induced a robust proliferative host cell response peaking at 24 hours post-infection (Fig 5). This UPEC-induced host cell proliferation was significantly blocked by UNC1999 (Fig 5). There was a correlation between EZH2 and PCNA expression in the infected groups by Pearson’s correlation analysis (R^2^ = 0.718, S2 and S3 Figs), but the correlation was not significant in the uninfected groups.

**Fig 6:**
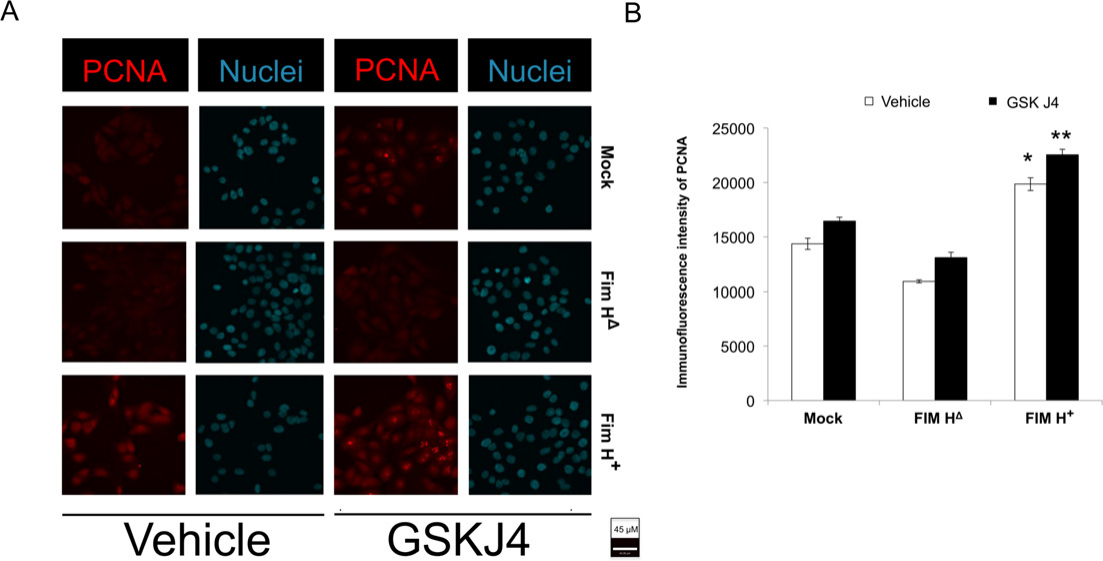
Inhibition of H3K27 demethylation increases the basal and infection-induced proliferation. (A) Immunofluorescence of PCNA was performed to test for proliferation of HT-5637 cells 24 hours after inoculation with vehicle, Fim H^+^ or Fim HΔ. As cell numbers vary during early after inoculation due to apoptosis and cell lysis, cell numbers were not counted to assay proliferation. Instead, proliferation was detected by PCNA staining. The highly selective inhibitor of histone K27 demethylases (KDM6A and B), GSK J4 (100 nM), enhanced urothelial proliferation in response to infection at 24 hours post-inoculation (p.i.). (B) Fluorescent signal for PCNA was acquired by confocal microscopy and quantitated on Volocity software. PCNA was significantly increased by UPEC Fim H^+^ bacteria inoculation of host cells (p<0.000001 vs. other groups). Demethylase inhibition with GSK J4 increased proliferation compared to corresponding vehicle treatment groups (* **vs.**, **, p<0.001,). N = 8.

Lysine(K)-specific demethylases 6A and B (KDM6A and B) act in the opposite manner to EZH2, as they catalyse **de**methylation at histone 3 lysine 27. We then tested if the inhibitor of KDM6A and B (GSK J4) had the opposite effect to EZH2 inhibition with UNC1999. Indeed, GSK J4 caused an increase in post-infection and basal levels of proliferation (Fig 6). The above inhibition of proliferation by UNC1999 and promotion of proliferation by GSKJ4 are complementary evidence supporting a role for H3K27 trimethylation in the proliferative response to UPEC infection. To our knowledge, this is the first demonstration of the functional role of a host cell epigenetic writer in infection-induced proliferation.

### UPEC infection-induced *WNT5A* expression depends on EZH2

**Fig 7:**
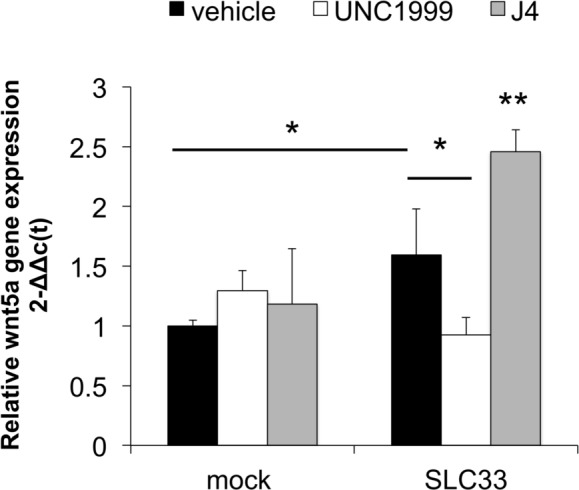
H3K27 trimethylation regulates *WNT5A* expression in response to UPEC infection. QPCR (^**ΔΔ**^c(t) method) was performed to detect EZH2 vs. reference gene expression. UPEC infection induced expression of *WNT5A* at 18 hours post-inoculation with 2 moi of UPEC Fim H^+^ bacteria compared to mock infected cells (*, p<0.05). The UPEC-induced *WNT5A* expression was reduced by treating with the EZH2 inhibitor UNC1999 (*,p<0.05) and increased further with the KDM6A/B inhibitor GSK J4 (**, vs. all other columns, p<0.05). N = 5.

*WNT5A* has been reported by others as a regulator of epithelial and uroepithelial cell growth during regeneration as well as post-infection. We therefore queried whether *WNT5A* was regulated alongside UPEC proliferation by EZH2. We found that UPEC lead to elevated *WNT5A* expression in the host cells (Fig 7, *p*<0.05). This increase in *WNT5A* was dependent upon EZH2 activity, as UNC1999 decreased UPEC-induced *WNT5A* mRNA. Inhibition of the demethylation at H3K27 by GSK J4 led to the opposite expression pattern with a significant increase in the *WNT5A* in the infection group. This dependency of *WNT5A* expression on EZH2 during infection has not been previously noted.

## Discussion

Intracellular pathogens and their host cells interact through signaling and secreted factors in both extracellular and intracellular environments. Here, we demonstrate that a major host epigenetic writer EZH2 is upregulated between 18–24 hours after inoculation with uropathogenic *E*. *coli* (UPEC). This upregulation correlates with an EZH2-dependent proliferative response to UPEC occurring 24 hours post-inoculation. We also show that this intracellular pathogen, which does not come into contact with urothelium except in the case of urinary tract infection (UTI), induces secretion of paracrine factors, including extracellular vesicles (EV), by infected host cells with intact activity to increase EZH2 in naïve cells.

Previously, we reported on the epigenetic regulation of a growth regulatory gene (CDKN2A) by DNA hypermethylation at 6 days post-inoculation. In this report, we found a biological response to infection, acute proliferation at 24 hours, which was mediated through activity of an epigenetic writer, EZH2. Direct evidence for EZH2 activity occurs through the increase in H3K27me3 levels in the host cells, similar to another study[51]. Many reports show that EZH2 plays a central role in proliferation through both regulation of proteins involved in proliferation as well as downregulation of expression of several tumour suppressor genes [28,29,36,52,53]. In contrast to the inhibition of H3K27 trimethylation, inhibition of H3K27 *de*methylation leads to increased proliferation consistent with a role of the H3K27me3 mark in controlling proliferation. Whether EZH2-dependent reductions of CDKN2A products (p16 and alternate reading frame (ARF)) or other EZH2-dependent mechanisms lead to the proliferative changes in post-infection remains under current investigation. Other transcriptional mechanisms are hinted at through the involvement of EZH2 in proliferation and Wnt signaling in intestinal cells during *Citrobacter* infection [36]. This intestinal response was associated with EZH2’s repression of Wnt inhibitory factor (WIF1) which releases inhibition of Wnt/beta-catenin. In another type of urinary tract infection, schistosomiasis, DNA methylation of WIF1 was found in human and mouse samples. In our model, UPEC infection increases *WNT5A* expression, which can increase Wnt signaling. Interestingly, during *Citrobacter* infection, rescue of WIF1 expression with EZH2 SET domains, did not entirely return downstream WNT signaling (as evidenced by nuclear β-catenin) to normal levels [36]. The lack of complete rescue may derive from persistence of other forms of epigenetic repression such as DNA methylation, which can is also seen in bladder schistosomiasis. However, our observed increase in *WNT5A* mRNA during infection was reversed with the inhibitor of EZH2 suggesting that a transcriptional repressor may be regulated by EZH2 in this case. Direct relationships amongst WNT expression and signaling, EZH2 and urinary tract infection-induced proliferation remains an exciting area to be explored in greater depth. However, both persistent and short-term epigenetic events may occur during UPEC in vitro infection, as the EZH2 and CDKN2A DNA methylation data reveals.

Our model allows for both persistent intracellular infection of the host (S5 Fig) and host survival. FimH^+^ bacteria increase the level of intracellular infection in turn increasing the EZH2-dependent proliferative response of the host cells. Nevertheless, the EZH2-dependent proliferative response to FimH^+^ UPEC could have either a protective or pathogenic role. *In vivo*, urothelial proliferation post-inoculation is initially protective as it replaces cells lost during initial desquamation or shedding of infected urothelial cells during fulminant infection. In vitro, a previous report showed that an inoculum of 2 moi of UPEC/host cell allowed for continued detection of the intracellular bacteria over the six day culture period, at relatively low numbers per cell[11]. This indicates that this low level of moi may allow for persistence, in contrast to the host cell lysis or cell death that results from high moi infection in vitro with 15–200 moi/host cell[27,54]. Signaling through paracrine factors in CM or EV to neighbouring uninfected urothelial cells may induce a proliferative response to aid in host recovery from primary infection. However, this response might also benefit the pathogen, as active host cell proliferation may create a beneficial host environment to harbor and disseminate intracellular pathogens. Consistent with this, urothelial cells *in vitro* treated with EV from infected cells respond with an altered morphology (smaller cell size with less cytoplasm) suggestive of a less differentiated phenotype (Fig 3).

EV and the conditioned media from infected cells may contain many factors, protein, mRNA and a plethora of miRNA species can induce alterations in recipient cells[11,22–24,26,38,55–58]. Of the EV, exosomes are very small vesicles, most between 30 and 150 nm, that allow for intercellular communication and the influencing of gene expression patterns[38–42,45]. In the intestine, a protozoal infection led to increased release of EV from host cells compared to uninfected controls[3], similar to the qualitative increase in EV release after UPEC infection seen here (Fig 2). Interestingly, these protozoal-induced exosomes were released in response to signaling from TLR4[3], which also acts as the receptor for Fim H and LPS of UPEC[17,59]. The protozoal-induced EV carry a cargo of antimicrobial peptides (AMPs) in particular beta-defensin2 and cathelicidin-37), which act as host protective factors. Protection against UTI has also been associated with presence of AMPs such as beta-defensins-2,3 and 14 I[18,60]. In the protozoal infection work, however, control exosomes derived from uninfected cells were not examined. In our study, the control EVs appear to decrease EZH2 levels in contrast to both FimH+ and FimH^**Δ**^-induced EV. It may be of interest to note that TLR4 is the receptor for both LPS and Fim H, and could be stimulated by Fim H^**Δ**^ mutant strains to through LPS instead of FimH. This could explain the Fim H^**Δ**^ –EV-induced rise in EZH2 compared to the basal levels or the mock-EV-induced decrease in EZH2. Nevertheless, as the Fim H^**+**^–EV and Fim H^**Δ**^ UPEC are able to induce EZH2 but FimH^**Δ**^ UPEC do not induce proliferation, it would appear that EZH2 is necessary but not sufficient to induce the proliferative response seen with FimH^+^ UPEC. In our ongoing and future studies, we hope to delineate the factors from EV derived from both infected and uninfected cells that regulate EZH2 and the pathobiology of urinary tract infection.

In summary, a critical epigenetic mediator of proliferation, EZH2, is increased in activity and expression by both direct infection and infection-induced paracrine host factors. The alteration of naïve cells by paracrine host factors as well as by direct infection has potential for effects on neighbouring naïve and/or intracellularly infected cells. We also saw the dependency of the proliferative and transcriptional responses to intracellular uropathogenic *E*.*coli* infection on EZH2. Future work will examine the *in vivo* importance of this epigenetic writer in the homeostasis of the urothelium post-infection, as epigenetic responses appear to play a role in proliferation of cultured urothelial cell in the face of a primary infection.

## Supporting Information

S1 Fig **Cell viability of HT-5637 urothelial cells to varying doses of EZH1/2 and KDM6A/B inhibitors, UNC1999 (Figures A and B) and GSK J4 (Figure C), respectively.** Cell viability was assayed by detecting resorfin production from raszurin, as measured on a fluorescent plate reader. Dosages in the range that did not affect basal viability of the cells were selected for use in Figs 5–7. n = 8.
(PDF)

S2 Fig EZH2 and PCNA expression increased at day one post-inoculation in HT-5637 cells. 10^5^ host cells were inoculated with 2 moi of uropathogenic *E*.*coli* (UTI89). Immunofluorescent staining was performed at 0, 1, 2 and 3 days post-inoculation. N = 8.
(PDF)

S3 Fig EZH2 and PCNA expression increase at day one post-inoculation in HT-5637 cells. 5X10^5^ host cells were inoculated with 2 moi of uropathogenic *E*.*coli* (UTI89). Immunostaining was performed at 0, 1, 2 and 3 days post-inoculation. N = 8.
(PDF)

S4 Fig UNC1999 was well tolerated by host cells. **Figure A**. Cell viability at day 2 was not significantly altered by UNC1999 treatment. **Figure B**. EZH2 expression was not altered by UNC1999 treatment at 2 day post-inoculation in HT-5637 cells. 10^5^ host cells were inoculated with 2 moi of uropathogenic *E*.*coli* (UTI89). QPCR was performed on samples harvested 2 days post-inoculation. N = 8.
(PDF)

S5 Fig FimH^+^ UPEC mutants demonstrated intracellular persistence at 6 days post-inoculation in HT-5637 cells cultured in Media with Gentamycin. FimH^+^ and FimH^**Δ**^ complemented SLC2 derivatives of UPEC were detected by anti-*E*.*coli*-biotin LPS antibodies with Cy3-conjugated Streptavidin, with nuclear Hoechst 33342 dye shown. Images were acquired by confocal microscopy. N = 3. Representative photomicrographs shown.
(PDF)

S1 Table Primer Sequences for QPCR. (PDF)
