# Supplementary figures and images for: Uropathogenic E.coli (UPEC) Infection Induces Proliferation through Enhancer of Zeste Homologue 2 (EZH2)
Source: PLoS One. 2016 Mar 10;11(3):e0149118. doi: 10.1371/journal.pone.0149118 (PMC4786126; doi:10.1371/journal.pone.0149118)

S1 Fig

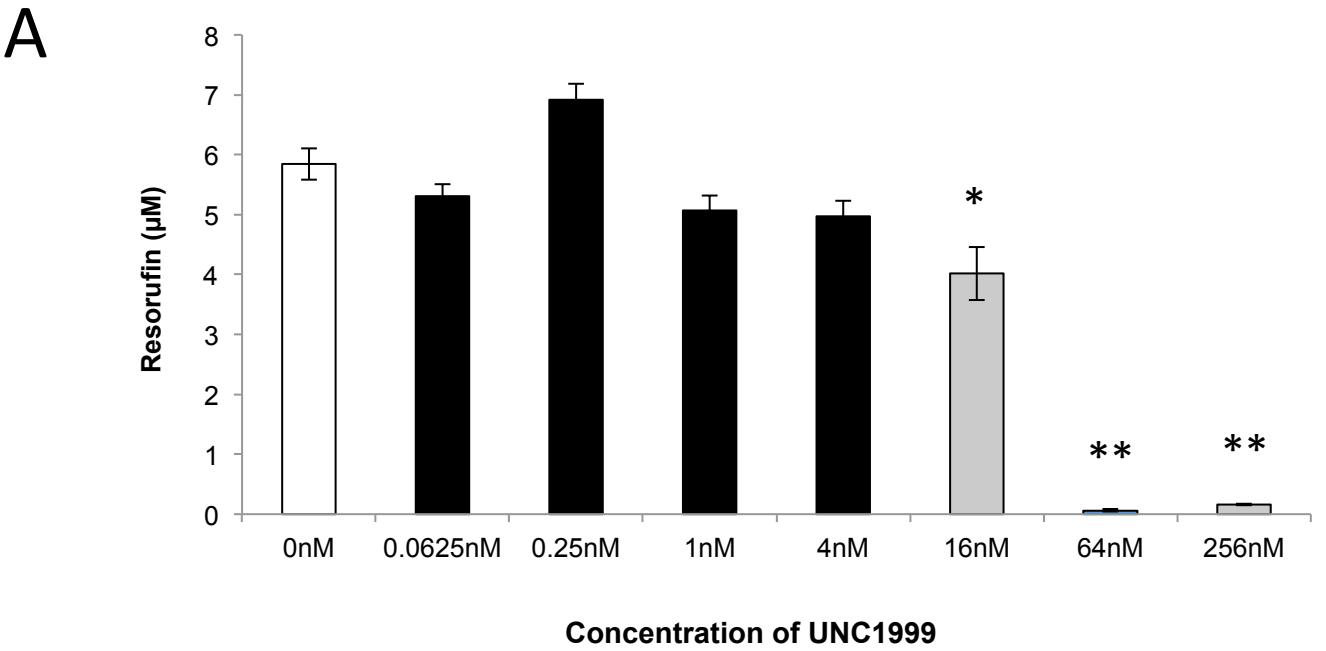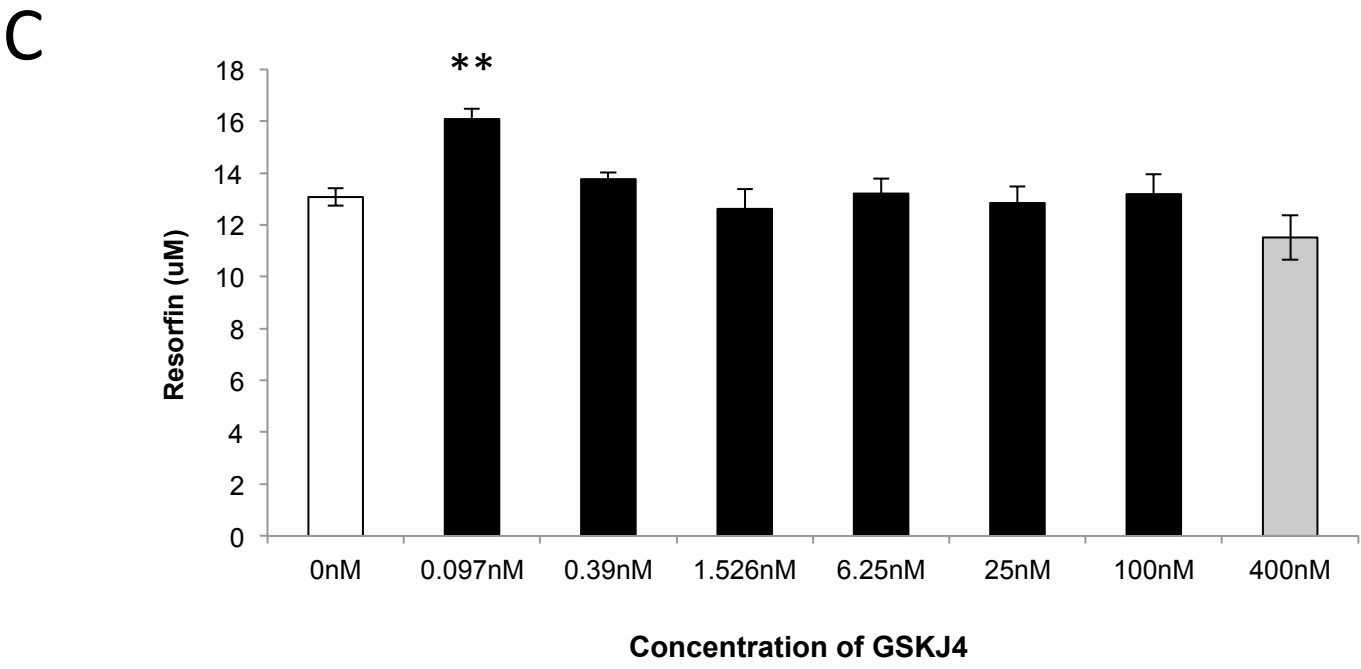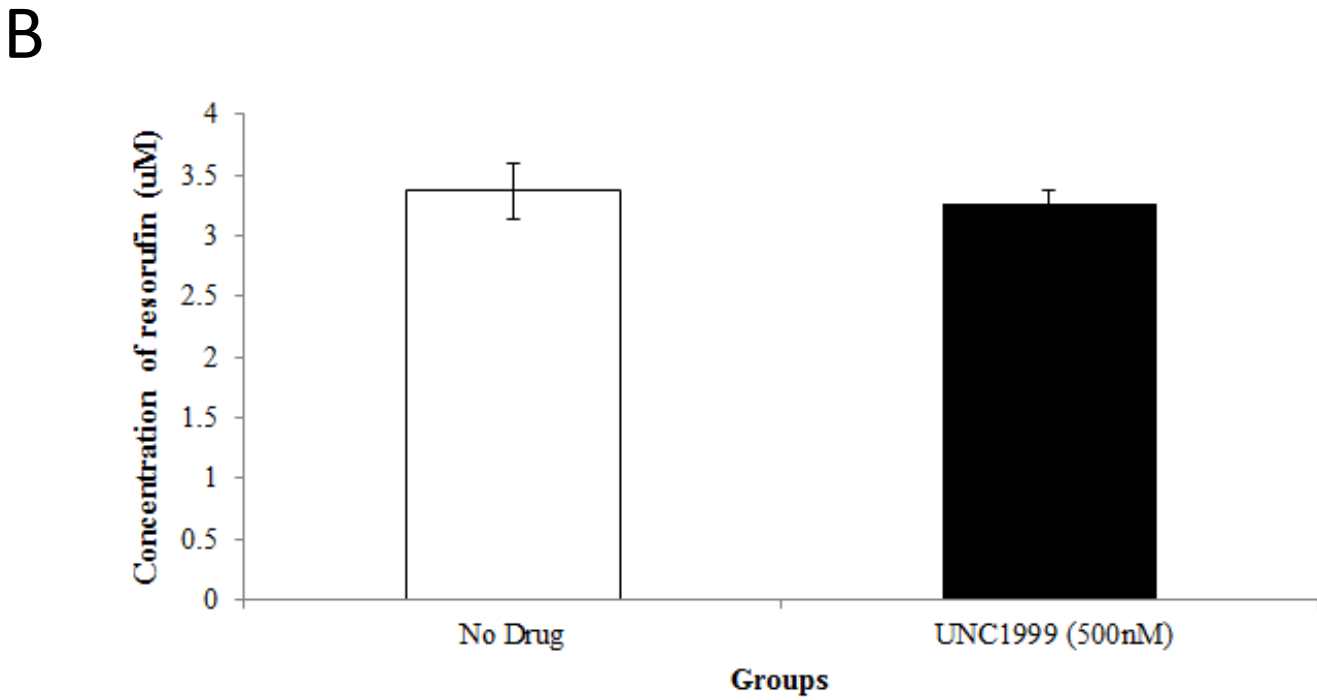

Supplement: S1 Fig — Cell viability of HT-5637 urothelial cells to varying doses of EZH1/2 and KDM6A/B inhibitors, UNC1999 (Figures A and B) and GSK J4 (Figure C), respectively. Cell viability was assayed by detecting resorfin production from raszurin, as measured on a fluorescent plate reader. Dosages in the range that did not affect basal viability of the cells were selected for use in Figs 5–7. n = 8. (PDF) [file pone.0149118.s001.pdf]

S2 Fig

A

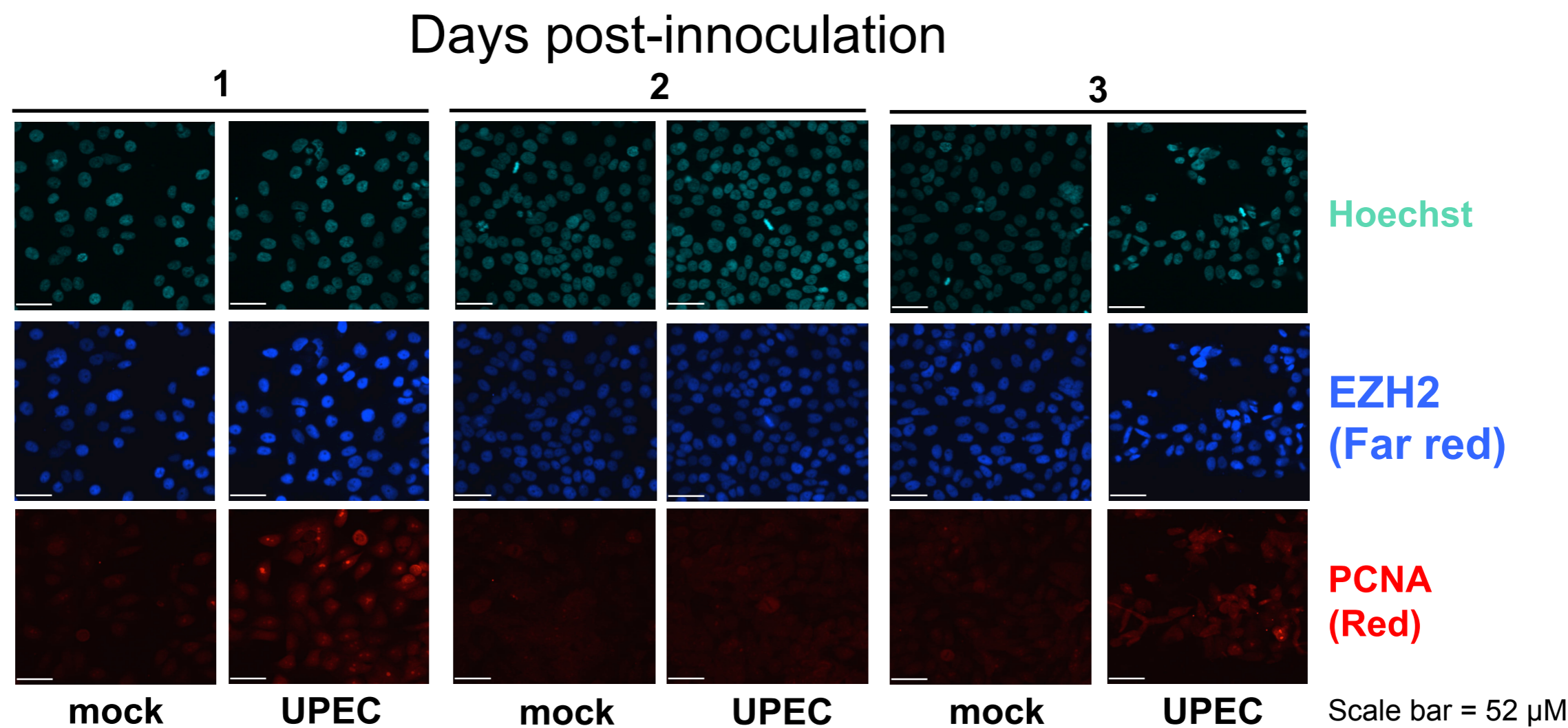

B

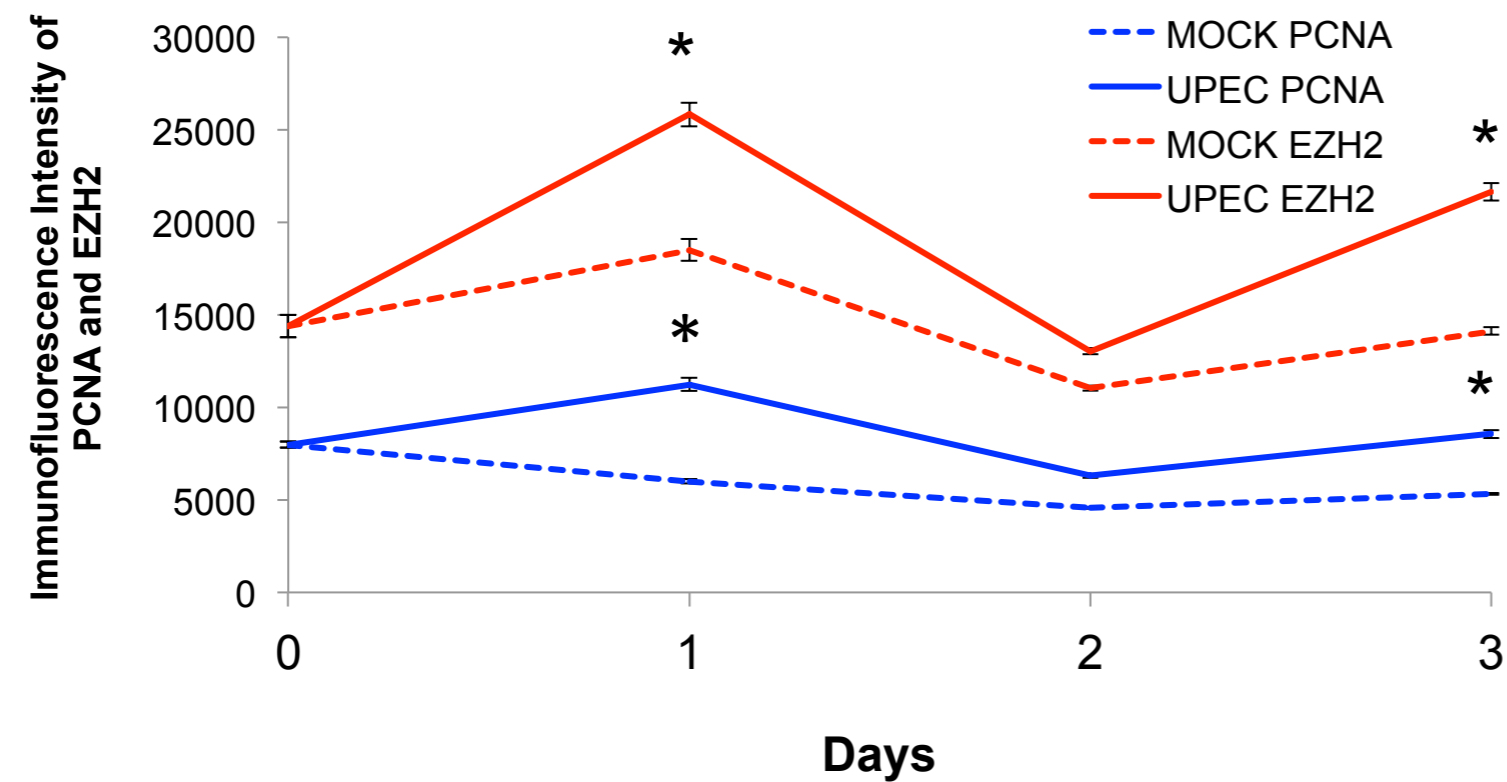

Supplement: S2 Fig — 105 host cells were inoculated with 2 moi of uropathogenic E.coli (UTI89). Immunofluorescent staining was performed at 0, 1, 2 and 3 days post-inoculation. N = 8. (PDF) [file pone.0149118.s002.pdf]

S3 Fig

Days post-innoculation

A

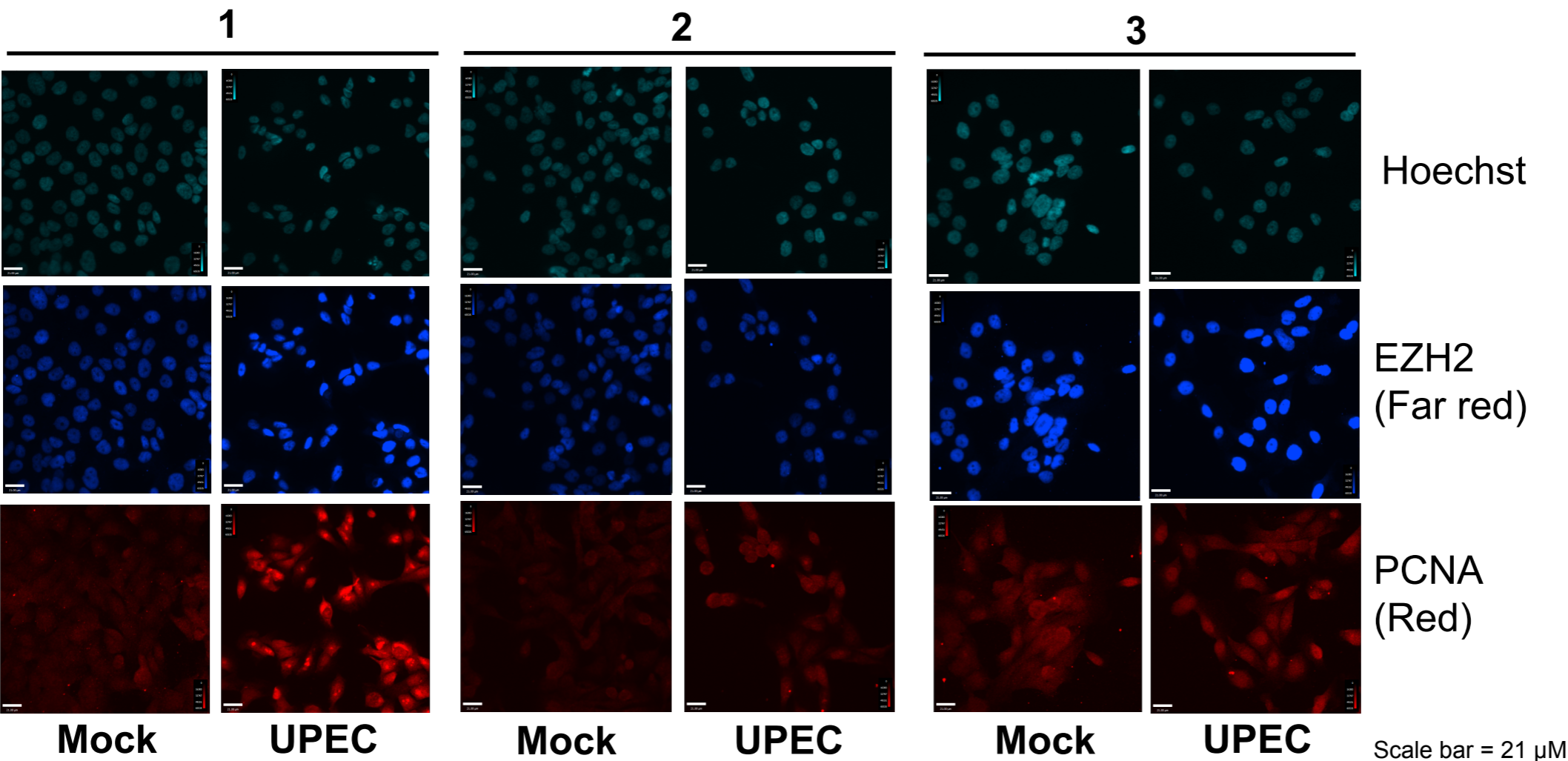

B

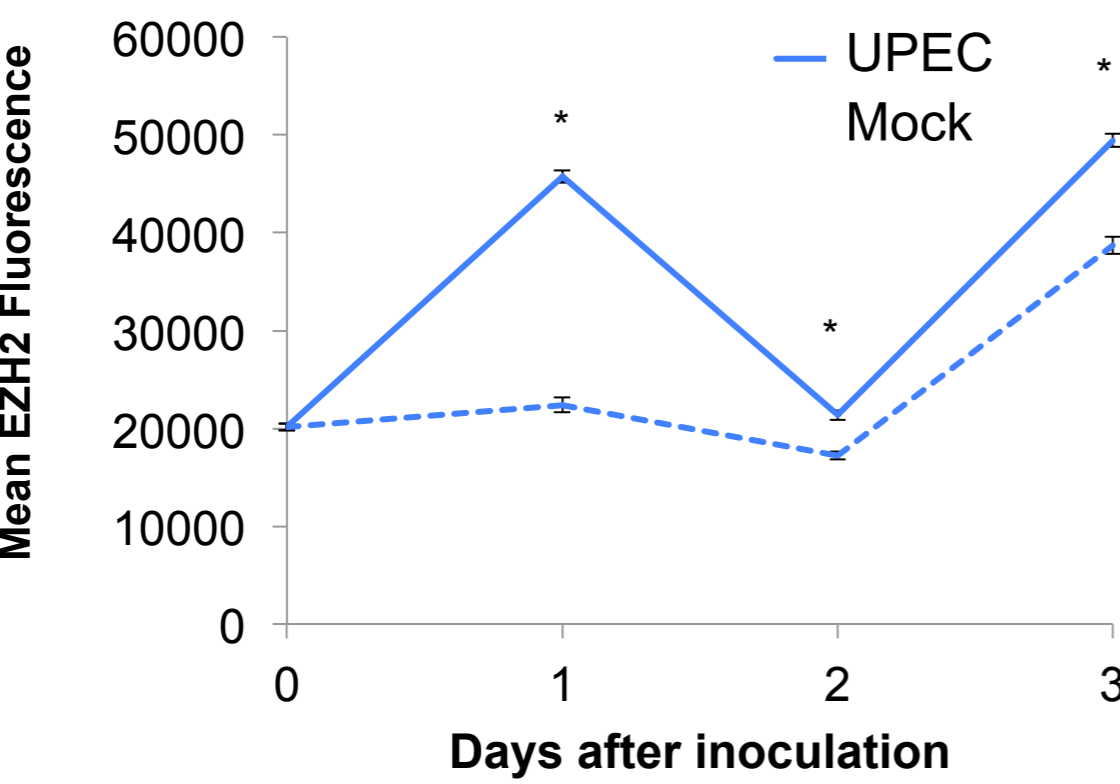

C

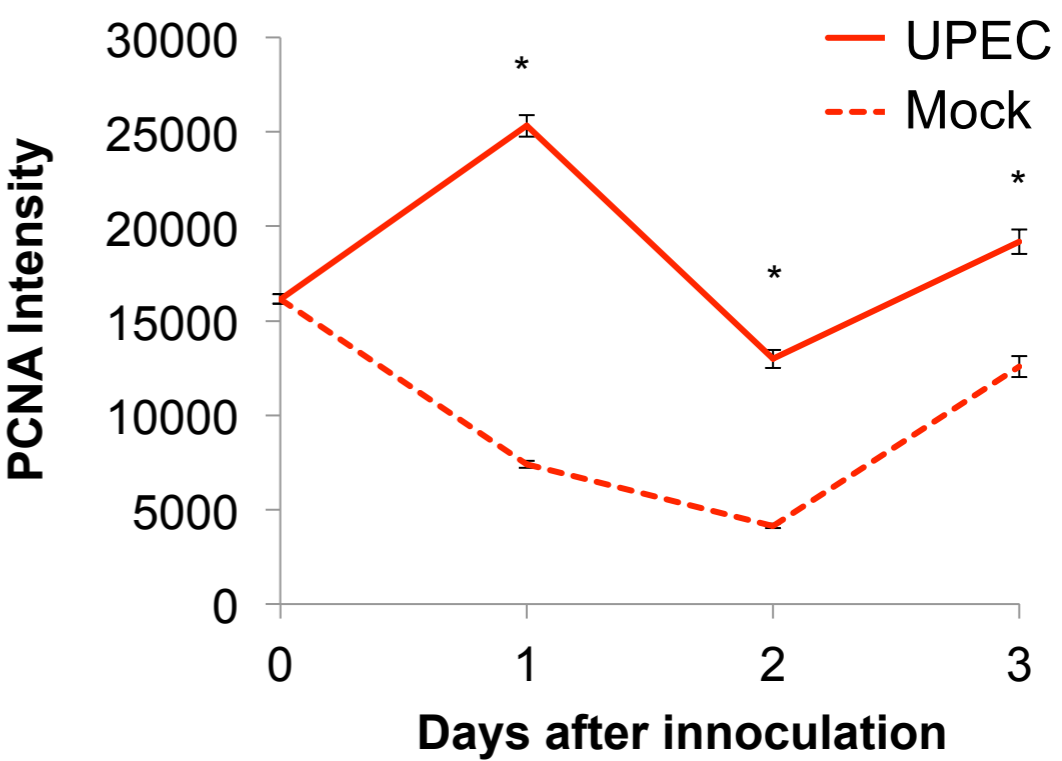

Supplement: S3 Fig — 5X105 host cells were inoculated with 2 moi of uropathogenic E.coli (UTI89). Immunostaining was performed at 0, 1, 2 and 3 days post-inoculation. N = 8. (PDF) [file pone.0149118.s003.pdf]

# S4 Fig

A 24 hours

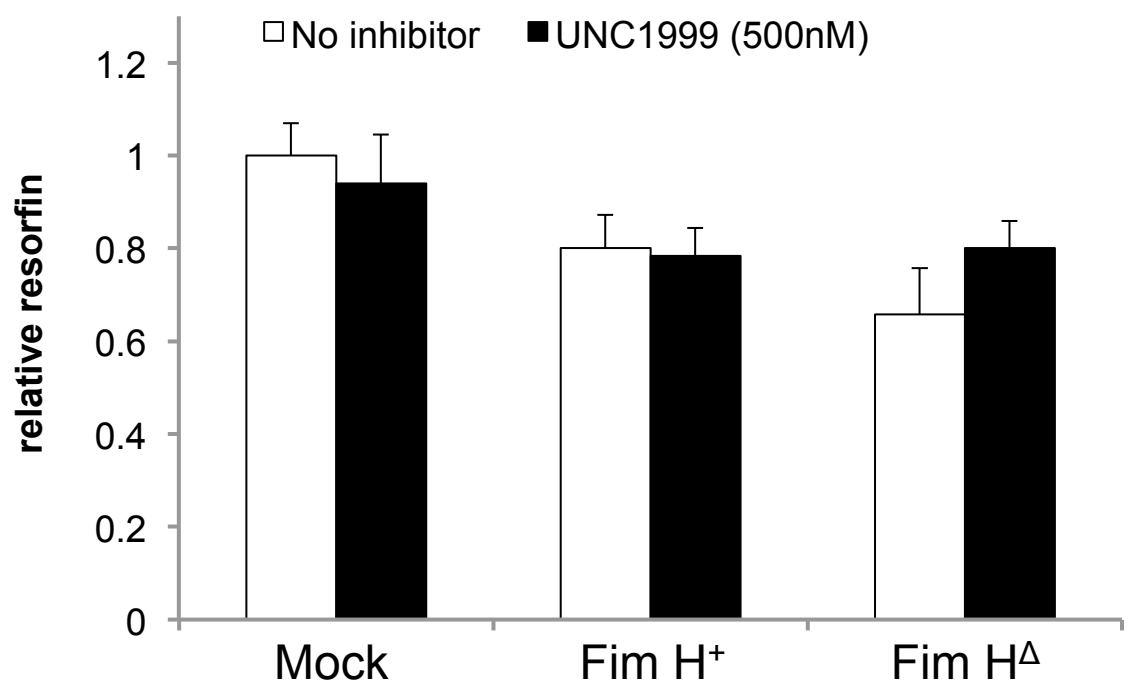

B 48 hours

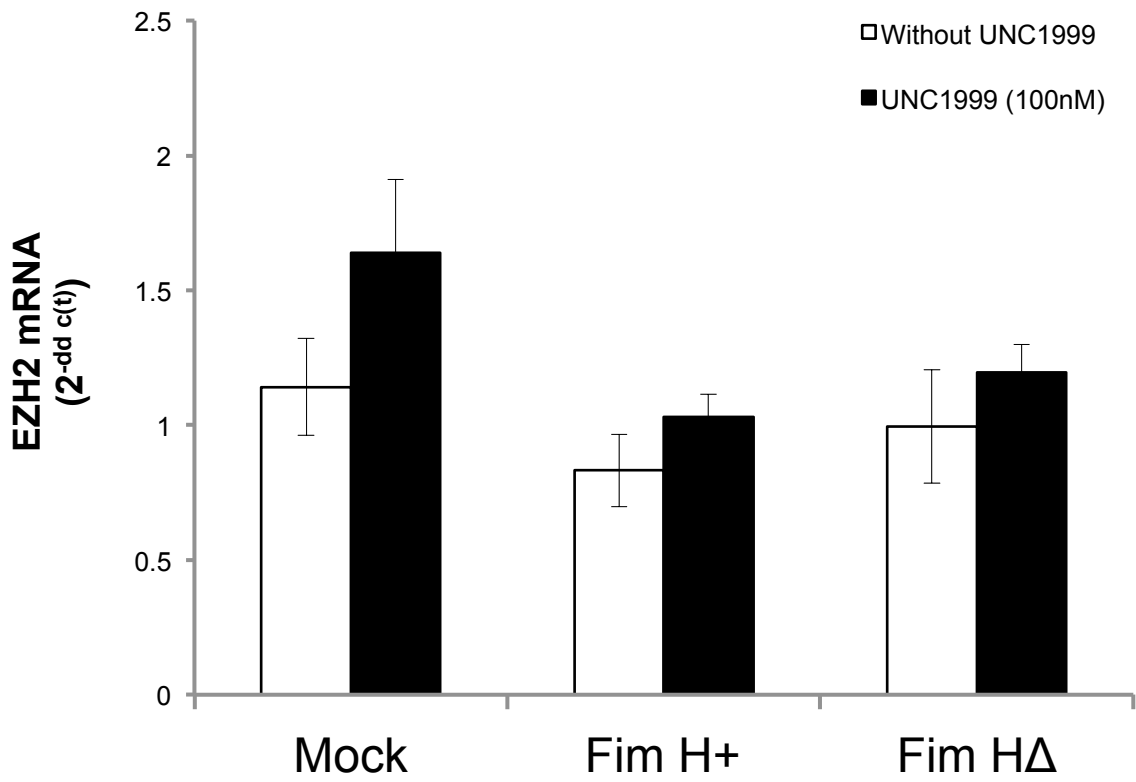

Supplement: S4 Fig — Figure A. Cell viability at day 2 was not significantly altered by UNC1999 treatment. Figure B. EZH2 expression was not altered by UNC1999 treatment at 2 day post-inoculation in HT-5637 cells. 105 host cells were inoculated with 2 moi of uropathogenic E.coli (UTI89). QPCR was performed on samples harvested 2 days post-inoculation. N = 8. (PDF) [file pone.0149118.s004.pdf]

## S5 Fig

No bacteria  
(mock)

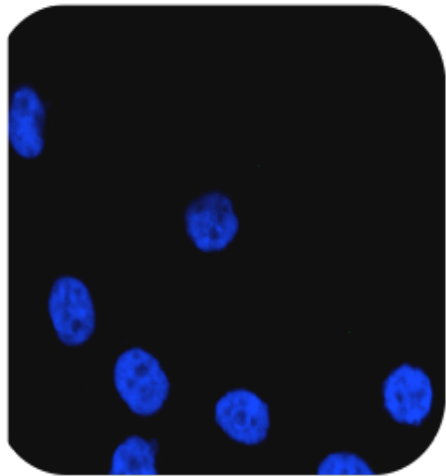

*UPEC (FimH+)*

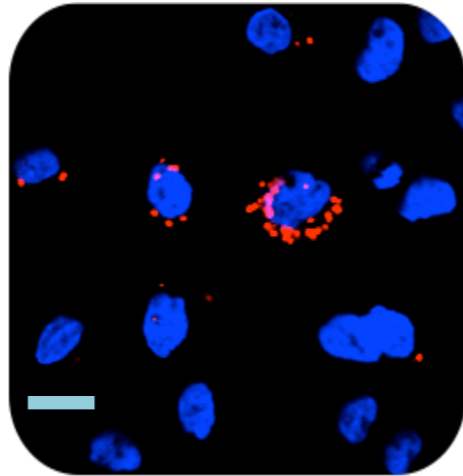

*Non-UPEC  
(FimHΔ)*

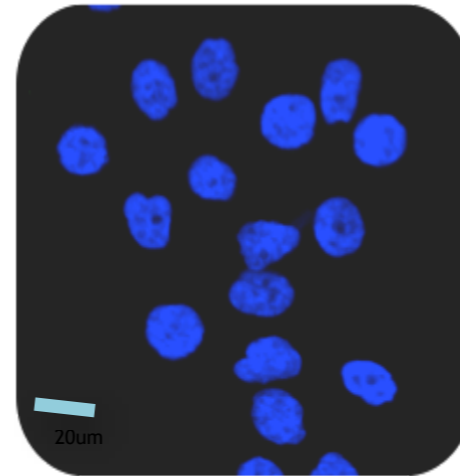

Nuclei

*E. Coli* LPS

Supplement: S5 Fig — FimH+ and FimHΔ complemented SLC2 derivatives of UPEC were detected by anti-E.coli-biotin LPS antibodies with Cy3-conjugated Streptavidin, with nuclear Hoechst 33342 dye shown. Images were acquired by confocal microscopy. N = 3. Representative photomicrographs shown. (PDF) [file pone.0149118.s005.pdf]
