# Supplementary material for: Uropathogenic E.coli (UPEC) Infection Induces Proliferation through Enhancer of Zeste Homologue 2 (EZH2)
Source: PLoS One. 2016 Mar 10;11(3):e0149118. doi: 10.1371/journal.pone.0149118 (PMC4786126; doi:10.1371/journal.pone.0149118)
Supplement: S1 Table — (PDF) [file pone.0149118.s006.pdf]

S1 Table

Gene	Forward Primer	Reverse Primer	Tm ($_{0}$C)	Product Size (bp)
GAPDH	GTC AGT GGT GGA CCT GAC CT	TGC TGT AGC CAA ATT CGT TG	56	147
EZH2 – isoform b	GCT TCC TAC ATC GTA AGT GCA A	CTC CCT CCA AAT GCT GGT AAC	61	125
Wnt5a	CTT CGC CCA GGT TGT AAT TGA AGC	CTG CCA AAA ACA GAG GTG TTA TCC	61	273
RPL19	CCA CAT GTA TCA CAG CCT GTA	CTT GGT CTT AGA CCT GCG G	57	153
